# Factors associated with HIV infection among men who have sex with men in Henan Province, China: a cross-sectional study

**DOI:** 10.1186/1471-2458-13-356

**Published:** 2013-04-17

**Authors:** Jie Liu, Bo Qu, Ezeakile Moses C, Yang Zhang, Shijie Liang

**Affiliations:** 1 Faculty of Health Statistics, School of public health, China Medical University, 92 North Second Road, Shenyang 110001, P.R. China; 2 Center for Disease Control and Prevention, Zhengzhou 450000, China

**Keywords:** Risk factors, MSM, Sexual behaviors, HIV infection

## Abstract

**Background:**

HIV prevalence among men who have sex with men (MSM) has increased rapidly in China. Behavioral and biological interventions are key to controlling the spread of HIV in the MSM population and the primary strategy for reducing the spread of AIDS in China. The purpose of this study is to investigate the prevalence of HIV among MSM in Henan province and to assess their knowledge levels and risk behaviors related to HIV/AIDS.

**Method:**

A cross-sectional survey was conducted among 388 MSM in 2010 in Zhengzhou City, Henan province, China.

**Results:**

Of the 388 respondents, 13.1% were infected with HIV and 10.3% were infected with syphilis. The results of multivariate analysis showed that participants who had a history of being infected by syphilis were more than 4 times more likely to be HIV positive (AOR=4.91; 95% CI: 1.70 to 12.02). For those who were residents from other provinces, the risk of HIV infection was 5.53 times higher (OR=5.53, 95% CI: 1.14, 6.25). Receipt of condoms (AOR = 0.15; 95% CI: 0.02 to 0.87), consistent condom use during last intercourse with a male (AOR=0.35; 95% CI: 0.14 to 0.87), and consistent condom use during last intercourse with a female (AOR=0.16; 95% CI: 0.08 to 0.90) were associated with a lower risk of HIV infection.

**Conclusion:**

The study suggests that some intervention strategies, including education intervention, condom promotion and distribution, and HIV counseling and testing are necessary to control HIV infection among MSM.

## Background

Although some HIV prevention interventions have been conducted in the past two decades, China has still entered a critical stage of rapid and widespread increase of the HIV/AIDS epidemic [1,2]. By 2007 the major groups infected with HIV were injection drug users (IDUs; 38.5%), former blood donors (FPDs; 19.3%) and promiscuous heterosexuals (17.8%) [3]. In 2009, sexual transmission, including both heterosexual and homosexual transmission, has become the dominant route of HIV infection, accounting for more than 70% of the estimated new infections in China [4]. Of particular concern is the increasing HIV prevalence among men who have sex with men (MSM). The percentages of newly reported HIV cases attributable to MSM in China were 0.2% in 2001, 7.3% in 2005, 12.2% in 2007, and 32.5% in 2009 [5].

MSM are disproportionately affected by the HIV pandemic due to social and behavioral factors that increase their vulnerability to HIV infection. Evidence from studies show that MSM are at a high risk for HIV infection in China also because of their high number of different sexual partners and active sexual activities without protection [5]. The traditional Chinese culture does not openly endorse MSM behaviors, and there is strong social pressure against such behavior [6]. Under social pressure, many MSM marry in order to hide their sexual orientation. Therefore, MSM may play a bridging role in the spread of HIV and other STDS from a high-risk population, such as their male sexual partners, to the general population, such as their wives [7-10].

Additionally, high migration rates has been associated with higher risk for HIV infection in MSM [11]. Massive internal migration from rural areas to urban cities, especially large sized and medium-sized cities, resulted in MSM being more socially connected [12,13]. Zhengzhou, located in the center of China, is an important transportation hub. There are 223 million in the floating population in 2010. It has been broadly reported in rural regions of China that most MSM had sex with casual sexual partners and never used a condom [14]. Therefore, health interventions to reduce high-risk behavior are key to controlling the spread of HIV in the floating population.

The purpose of the study was to identify factors associated with HIV infection among MSM in China, in order to make primary strategies to control the HIV spread in MSM population.

## Methods

### Respondents and procedures

A cross-sectional study was conducted in Zhengzhou city, Henan Province, China, from May to June, 2010. Respondents were recruited through internet advertisement two weeks prior to the study, whereby they met with the trained researcher at various venues including gay bars, gay saunas, and parks where MSM meet one another. The respondents underwent a face-to-face explanation to a standardized questionnaire (Additional file 1), which was initially drafted based on the contents formulated by relevant previous studies [15,16]. All interviews were self-administered in a private room in the Center for Disease Prevention and Control (CDC) of Zhengzhou.

The questionnaire was divided into two parts. The first part included socioeconomic characteristics, such as age, marital status, and education, and the second part was about knowledge, service utilization and sexual-related behaviors. HIV/AIDS-related knowledge was measured with an 8-item set of questions covering the basic characteristics of the disease, routes of viral transmission and measures of prevention. Service utilization was measured with four items. Each item was attached to a two-point response scale: “yes” or “no”. Respondents were asked whether they had ever received Voluntary Counseling and Testing (VCT) services and whether they had utilized various types of HIV-related services in the past 12 months. Sexual behavior was measured with nine items. Respondents were asked if they had used a condom in the past 6 months and the frequency of use when they had sex with a male or female or when they had commercial sex with a male partner. Condom use in the past six months was attached to a two-point response scale: “yes” or “no”. The frequency of condom use was attached to a three-point response scale with scores that ranged from “1” for “always” to “3” for “seldom”.

Before formal investigation, the interviewers had been trained together to make them understand the purpose and meaning of the investigation and to be familiarized with the contents of the questionnaire. They were also trained to explain any question in the questionnaire on the spot. A verbal informed consent was obtained from each respondent before the survey. Participation in the study was completely voluntary and the respondents had the option of declining to answer specific questions or of leaving the entire questionnaire blank if they did not wish to participate. After completion of the questionnaires, specially trained personnel inspected the questionnaires, identified the questionnaires that were filled out with non-standard and ambiguous answers and found investigators and respondents for timely verification. All participants provided blood specimens, which were tested for both HIV and syphilis antibodies. HIV antibodies were detected by enzyme-linked immunosorbent assay (ELISA) (Beijing Wantai Biologic Medicine Co., China). Reactive ELISA specimens were confirmed by a HIV-1/2 Western Blot immune assay (HIV Blot 2.2 WB; Genelabs Diagnostics, Singapore). Samples testing positive in both tests were considered HIV-positive. Syphilis antibodies were detected by ELISA (Beijing Jinhao Biologic Production Co., China). Collected specimens were tested by the Center for Disease Prevention and Control of Zhengzhou. An incentive of 100 Yuan (equivalent to about 15 USD) was given to respondents as compensation for the time spent being interviewed. The study protocol was approved by the bioethics advisory commission of the China Medical University.

### Data analysis

Data were double entered and evaluated for congruency using the EpiData software (version 6.4; EpiData Association; Odense, Denmark). Descriptive analyses were conducted to describe the socioeconomic characteristics of the respondents, sexual behaviors and condom use, HIV/AIDS knowledge and social service utilization, prevalence of HIV and syphilis, and the risk factors for HIV infection. Univariate and multivariable logistic regression analyses were performed using the SPSS software (Version 16.0; SPSS Inc, Chicago, IL) to identify factors associated with HIV infection. Variables with a p-value < 0.10 at univariate results were considered eligible for the multivariate analysis. A backward procedure based on the Wald test was used to select significant variables in the multivariate model. The significance level was fixed at α=0.05.

## Results

### Socioeconomic characteristics

A total of 404 MSM were surveyed for the study. In total, 388 MSM completed the questionnaire (response rate: 96.0%). The ages of respondents ranged from 18 to 73, with a mean age of 31.7 years. 298 (76.8%) of respondents were local residents of the study site, 23.2% lived in a different province. The majority (81.4%) belongs to the Han ethnic group. 49.2% of the respondents self-identified as bisexual. 44.6% of respondents were single, 46.6% were married, and 8.8% were divorced or widowed. The age-specific prevalence of marital status was as follows: less than 30 years old (17.4%), more than 30 years old (71.4%), and more than 40 years old (80.6%). 67.0% lived in Zhengzhou for more than 1 year, 13.9% for 3 months to 1 year, and 19.1% for less than 3 months. (0.5%) participants in our study used illicit drugs in the past 6 months.

### Sexual behaviors and condom use

Among the respondents, most (88.1%) reported having anal sex with a man in the past 6 months, and 33.6% reported never having used condoms in their last intercourse with a male partner. 17.8% reported having never used a condom during anal sex with a man in the previous 6 months. 49.2% reported having had sex with a woman in the past 6 months, and 30.4% of these reported never always using a condom. Additionally, 8.5% reported having paid sex with a man in the past 6 months, and 48.5% of these reported always having used a condom (Table 1).

**Table 1:** Sexual behaviors of MSM in Zhengzhou in 2010

	**Number**	**Percentage (%)**
Had sex with male in the past 6 months		
Yes	342	88.1
No	46	11.9
Used condom in the last sex with male		
Yes	227	66.4
No	115	33.6
Condom use during sex with male in the past 6 months		
Never	61	17.8
Sometimes	160	46.8
Always	121	35.4
Had commercial sex with male in the past 6 months		
Yes	33	8.5
No	355	91.5
Condom use during commercial sex with male in the past 6 months		
Never	3	9.1
Sometimes	14	42.4
Always	16	48.5
Used condom in the last commercial sex with male		
Yes	26	78.8
No	7	21.2
Had sex with female in the past 6 months		
Yes	191	49.2
No	197	50.8
Used condom in the last sex with female		
Yes	73	38.2
No	118	61.8
Condom use during sex with female in the past 6 months		
Never	58	30.4
Sometimes	107	56.0
Always	26	13.6

### HIV/AIDS knowledge and social service utilization

The majority (75.5%) of the respondents answered at least 6 of the 8 HIV-related questions correctly. A total of 90.5%, 90.5% and 85.3% of the subjects were aware that HIV was transmitted by “transfusions tainted with HIV”, “sharing intravenous needles” and “from mother to child”, respectively. The proportion of subjects who correctly selected non-transmission routes was low; e.g. “mosquitoes or other insects” (62.1%). 42.8% of the subjects were aware that a person with HIV can look as healthy as other people (Table 2). About half of the respondents received free condoms (61.6%) and peer education (53.9%), and one-third (30.4%) had received examinations for HIV in the past 12 months (Table 3).

**Table 2:** The HIV/AIDS related knowledge of MSM populations

**HIV/ AIDS transmission routes question**	**Yes**	**No**	**Unsure**
Might people who look healthy possibly carry HIV?	166 (42.8%)	163 (42.0%)	59 (15.2%)
Can HIV be spread by mosquitoes or other insects?	101 (26.0%)	241 (62.1%)	46 (11.9%)
Is it risky to eat with a person with HIV/AIDS?	50 (12.9%)	313 (80.7%)	25 (6.4%)
Could blood or blood product transfusions tainted with HIV cause infection with HIV?	351 (90.5%)	14 (3.6%)	23(5.9%)
Could sharing needles for drug use with someone who has HIV or AIDS cause HIV infection?	351 (90.5%)	10 (2.6%)	27 (7.0%)
Can a pregnant woman with HIV give the virus to her baby?	331 (85.3%)	30 (7.7%)	27 (1.1%)
Could correct use of condoms reduce the risk of HIV spreading?	340 (87.6%)	23(5.9%)	25(6.4%)
Could having a main sex partner reduce the risk of HIV spreading?	302 (77.8%)	46 (11.9%)	40 (10.3%)

**Table 3:** Factors associated with HIV infection among MSM in Zhengzhou

**Factors**	**Positive%**	**Total N**	**OR**^**1 **^**(95% CI)**	**AOR**^**2 **^**(95% CI)**
Age				
≤25(Ref)	12.0	125	1	
26-35	13.9	137	1.18(0.57, 2.44)	
>35	13.5	126	1.14(0.54, 2.41)	
Marital status				
Single (Ref)	12.1	173		
Married	15.5	181	1.05(0.22,4.88)	
Separated /divorced/widowed	5.9	34	0.65(0.36,7.50)	
Residency				
Local province (Ref)	10.1	298	1	1
Other province	23.3	90	2.72 (1.47, 5.05)*	5.53(1.14, 6.25)*
Ethnicity				
Han (Ref)	12.0	316	1	
Others	18.1	72	1.61 (0.81,3.21)	
Time of living in Zhengzhou				
<3 month (Ref)	18.9	74	1	
3 month-1 year	11.1	54	0.72 (0.66,3.41)	
>1 year	11.9	260	0.92 (0.37,2.34)	
Education level				
Junior high school or lower (Ref)	13.8	80	1	
Senior high school	15.8	158	1.44(0.63,3.29)	
College or above	10.0	150	0.30 (0.12,0.94)*	
appropriate responses to the 8 HIV-related knowledge items≥ 6				
No (Ref)	17.9	28	1	
Yes	14.6	314	0.67(0.24,1.89)	
Had sex with male in the past 6 months				
No(Ref)	8.7	46	1	
Yes	13.7	342	1.63(0.56,4.75)	
Condom use in the last sex with male				
No(Ref)	19.1	115	1	1
Yes	11.0	227	0.52(0.28,0.98)*	0.35(0.14,0.87) *
Condom use during sex with male in the past 6 months				
Never(Ref)	16.4	61	1	
Sometimes	15.0	160	0.94 (0.55,2.10)	
Always	10.7	121	0.27(0.03,2.12)	
Had commercial sex with male in the past 6 months				
No(Ref)	9.1	33	1	
Yes	13.5	355	1.56(0.46,5.32)	
Had sex with female in the past 6 months				
No(Ref)	15.2	197	1	
Yes	11.0	191	0.69(0.38,1.25)	
Condom use in the last sex with female				
No(Ref)	15.3	118	1	1
Yes	4.1	73	0.24(0.07,0.84)*	0.16 (0.08,0.90)*
Condom use during sex with female in the past 6 months				
Never (Ref)	13.8	58	1	
Sometimes	10.3	107	0.97 (0.24,2.73)	
Always	8.0	26	0.95 (0.23,4.01)	
Receipt of condoms				
No(Ref)	18.1	149	1	
Yes	10.0	239	0.50 (0.28,0.96)*	0.15 (0.02,0.87)*
Received peer education				
No(Ref)	14.5	179	1	
Yes	12.0	209	0.80 (0.41,1.43)	
Received HIV checks in the last year				
No(Ref)	13.3	270	1	
Yes	12.7	118	0.95(0.50,1.79)	
Diagnosed STDs in the last year				
No (Ref)	13.0	353	1	
Yes	14.3	35	1.05 (0.56,2.00)	
Syphilis status				
Negative(Ref)	11.5	348	1	1
Positive	27.5	40	2.92(1.34,6.30)*	4.91 (1.70,12.02)*

*:*p* < 0.05 OR = odds ratio; AOR adjusted odds ratio; CI = confidence interval;

STDs: sexually transmitted diseases.

### Factors associated with HIV infection

Of all the respondents, 13.1% (51) were infected with HIV and 10.3% (40) were infected with syphilis. The following factors were associated with higher risk of HIV infection by univariate analysis: being a nonlocal resident, lower levels of education, and syphilis infection. Receipt of condoms and consistent condom use in the last intercourse with either a male or female reduced the risk of HIV infection. Results of multivariate analysis showed that participants who had a history of being infected by syphilis were more than 4 times more likely to be HIV positive (AOR=4.91; 95% CI: 1.70 to 12.02). For those who were residents of other provinces in China, the risk of HIV infection was 5.53 times higher (OR=5.53, 95% CI: 1.14, 6.25). Receipt of condoms (AOR = 0.15; 95% CI: 0.02 to 0.87), consistent condom use during last intercourse with a male (AOR=0.35; 95% CI: 0.14 to 0.87), and consistent condom use during last intercourse with a female (AOR=0.16; 95% CI: 0.08 to 0.90) were associated with a lower risk of HIV infection. The results of univariate and multivariate logistic regression analysis are shown in Table 3.

## Discussion

In our study, 49.2% of the respondents self-identified as bisexual, about 46.6% of MSM respondents were married, and the prevalence of married MSM increased to 71.4% or greater among those MSM who were over 30 years old. In China, MSM are a hidden subgroup in mainstream society. Due to stigma, discrimination, denial and ignorance, it is a challenge to identify MSM. They may use marriage as a means to disguise their homosexual behaviors or sexual orientations. High-risk behaviors were common in this population. Only 66.4% of the respondents used a condom during their last sexual intercourse. Consistent condom use was low even with male sexual partners (35.4%). Moreover, the rate of consistent condom use with women was lower than with men. 49.2% of the respondents reported having had sex with women in the past 6 months, and 61.8% did not use a condom in their last sexual intercourse with a female. During sexual intercourse with a female in the past 6 months, most of the respondents sometimes used a condom, and 30.4% of the respondents had never used a condom. The high portion of men who have sex with both men and women could serve as a potential “bridge” for the spread of HIV and other STDs from the high-risk population to their female partners and to the general Chinese population.

Our study suggested that for respondents who were residents of other provinces, the risk of HIV infection was 5.53 times higher. Zhengzhou, the capital city of Henan province, is one of the major cities in middle China. Therefore, it has a much greater nonlocal population. It is difficult to get information to the nonlocal MSM population regarding social support. Our study found that the rates of HIV prevalence was higher than those reported in other districts in China [2,14,17,18]. Therefore, more effective strategies need to be implemented to control the HIV spread in nonlocal populations.

Studies have suggested a positive association between condom use in male-to-male anal intercourse and access to HIV preventives actions [19-26]. Our study also found that condom use in the last sexual intercourse were independently associated with HIV infection. In addition, we found accepting condoms significantly decreased the risk for HIV infection, which indicated that risky sexual behaviors associated with HIV infection could be constrained, in part, by increasing condom distributions. However, based on the results of our study, services utilization was still low in Zhengzhou. Only 30.4% of respondents reported having had an HIV test, and about 50% of respondents received condoms and peer education. Intervention programs for MSM, such as condom promotion and distribution and AIDS advisory and testing, and the distribution of materials on AIDS information need to be made more available and accessible to the MSM population.

Studies have demonstrated the connection between HIV and syphilis infections [27-30]. Our data also indicated a possible correlation between HIV and syphilis infections. After controlling for other risk factors, respondents who had been infected with syphilis were 4 times more likely to be infected with HIV. The high prevalence of syphilis suggested that MSM in Zhengzhou are at a greater risk for HIV infection. Widespread screening for syphilis infections in this risk group should be considered as a measure for control.

Unlike some other countries where drug use was considered as a major risk for HIV spread in the MSM population, only 2 participants in our study used illicit drugs in the past 6 months. It seems that there is little overlap of between the two populations of drug users and MSM in Zhengzhou.

The study has a number of limitations. First, the questionnaire data relying on retrospective self-reports was subject to recall bias. The findings should be interpreted carefully when generalizing the larger MSM population or comparing results from other studies. Second, our study was conducted in a limited area in Zhengzhou, China. Further work that focuses on extending the research into the broader areas in China would be valuable.

## Conclusion

Our study indicates that a tailored approach to HIV prevention among MSM in Henan province in China is urgently needed. Some intervention strategies such as education intervention, condom promotion and distribution, and HIV counseling and testing are necessary to control HIV infection among MSM.

## Competing interests

The authors declare that they have no competing interests.

## Authors’ contributions

LJ drafted the manuscript, participated in the collection of data, participated in the design of the study, and performed the statistical analysis. QB designed the study, participated in project coordination, and helped to draft the manuscript. MCE participated in the draft of the manuscript. ZY participated in the collection of data and performed the statistical analysis. LSJ participated in the collection of data. All authors read and approved the final manuscript.

## Pre-publication history

The pre-publication history for this paper can be accessed here:

http://www.biomedcentral.com/1471-2458/13/356/prepub

## Supplementary Material

Additional file 1 **MSM Questionnaire.** The MSM questionnaire included questions on socio-demographic characteristics, HIV/AIDS-related knowledge, service utilization and behaviors among MSM in Zhengzhou City. Click here for file
