# Supplementary material for: Factors associated with HIV infection among men who have sex with men in Henan Province, China: a cross-sectional study
Source: BMC Public Health. 2013 Apr 17;13:356. doi: 10.1186/1471-2458-13-356 (PMC3637619; doi:10.1186/1471-2458-13-356)
Supplement: Additional file 1 — MSM Questionnaire. The MSM questionnaire included questions on socio-demographic characteristics, HIV/AIDS-related knowledge, service utilization and behaviors among MSM in Zhengzhou City. [file 1471-2458-13-356-S1.doc]

**Questionnaire**

*一、Socioeconomic Characteristics*

1. Age: _____; 2.Date of birth:__________; 3. Ethnicity:________;

4. Education level:

① Junior high school or lower ② Senior high school ③ College or above

5. Marital status: ① Single ② Married ③Separated /divorced/widowed;

6. The age of marriage: _____;

7. Sexual orientation: ①Gay ② Bisexual

8. Residency: ① Local province ② Other province;

9. Time of living in Zhengzhou: _____;

10. Illicit drugs used: ① Yes ② No

11. Did you diagnose STDs in the last year: ① Yes ② No

12. Syphilis status: ① Negative ② Positive

二*、HIV/AIDS-related knowledge, service utilization and sexual-related behaviors*

1. HIV/AIDS Knowledge

| HIV/ AIDS transmission routes question |  |  |  |
| --- | --- | --- | --- |
| 1.1 Might people who look healthy possibly carry HIV? | ① Yes | ② No | ③ Unsure |
| 1.2 Can HIV be spread by mosquitoes or other insects? | ① Yes | ② No | ③ Unsure |
| 1.3 Is it risky to eat with a person with HIV/AIDS? | ① Yes | ② No | ③ Unsure |
| 1.4 Could blood or blood product transfusions tainted with HIV cause infection with HIV? | ① Yes | ② No | ③ Unsure |
| 1.5 Could sharing needles for drug use with someone who has HIV or AIDS cause HIV infection? | ① Yes | ② No | ③ Unsure |
| 1.6 Can a pregnant woman with HIV give the virus to her baby? | ① Yes | ② No | ③ Unsure |
| 1.7 Could correct use of condoms reduce the risk of HIV spreading? | ① Yes | ② No | ③ Unsure |
| 1.8 Could having a main sex partner reduce the risk of HIV spreading? | ① Yes | ② No | ③ Unsure |

2. Service Utilization

| 2.1 Received condoms | ① Yes | ② No |  |
| --- | --- | --- | --- |
| 2.2 Received peer education | ① Yes | ② No |  |
| 2.3 Received HIV checks in the last year | ① Yes | ② No |  |

3. Sexual-related Behaviors

| 3.1 Had you had sex with male in the past 6 months? | ① Yes | ② No |  |
| --- | --- | --- | --- |
| 3.2 Did you use condom in the last sex with male? | ① Yes | ② No |  |
| 3.3 How often had you used condom during sex with male in the past 6 months？ | ① Never | ② Sometimes | ③Always |
| 3.4 Had you had commercial sex with male in the past 6 months？ | ① Yes | ② No |  |
| 3.5 Did you use condom in the last commercial sex with male？ | ① Yes | ② No |  |
| 3.6 How often had you used condom during commercial sex with male in the past 6 months？ | ① Never | ② Sometimes | ③Always |
| 3.7 Had you had sex with female in the past 6 months？ | ① Yes | ② No |  |
| 3.8 Did you use condom in the last sex with female？ | ① Yes | ② No |  |
| 3.9 How often had you used condom during sex with female in the past 6 months？ | ①Never | ②Sometimes | ③Always |
